# Sarcopenia and osteoporosis are interrelated in geriatric inpatients

**DOI:** 10.1007/s00391-019-01553-z

**Published:** 2019-05-02

**Authors:** J. Reiss, B. Iglseder, R. Alzner, B. Mayr-Pirker, C. Pirich, H. Kässmann, M. Kreutzer, P. Dovjak, R. Reiter

**Affiliations:** 1 Department of Geriatric Medicine, Christian-Doppler-Klinik, Paracelsus Medical University Salzburg, Ignaz-Harrer-Straße 79, 5020 Salzburg, Austria; 2 Department of Endocrinology and Nuclear Medicine, Landeskrankenhaus, Paracelsus Medical University Salzburg, Müllner Hauptstraße 48, 5020 Salzburg, Austria; 3 Department of Clinical Nutrition, Christian-Doppler-Klinik, Paracelsus Medical University Salzburg, Ignaz-Harrer-Straße 79, 5020 Salzburg, Austria; 4 Salzkammergut-Klinikum Gmunden, Miller-von-Aichholz-Straße 49, 4810 Gmunden, Austria

**Keywords:** Osteosarcopenia, Muscle-bone-unit, Bone-muscle-continuum, Sarco-osteoporosis, Osteosarkopenie, Muskel-Knochen-Einheit, Knochen-Muskel-Kontinuum, Sarkoosteoporose

## Abstract

**Background:**

Sarcopenia and osteoporosis share an underlying pathology and reinforce each other in terms of negative outcomes.

**Objective:**

To evaluate the extent of concomitance of sarcopenia as defined by the European Working Group on Sarcopenia in Older People (EWGSOP) and osteoporosis as defined by the World Health Organization (WHO) in geriatric inpatients and their relationship to nutritional and functional status.

**Material and methods:**

A cross-sectional analysis of geriatric inpatients from the sarcopenia in geriatric elderly (SAGE) study. Measurements included dual X‑ray absorptiometry for bone mineral density and appendicular muscle mass; gait speed and hand grip strength, the Barthel index, body mass index (BMI) and the mini nutritional assessment short form (MNA-SF).

**Results:**

Of the 148 patients recruited for SAGE, 141 (84 women, 57 men; mean age 80.6 ± 5.5 years) had sufficient data to be included in this ancillary investigation: 22/141 (15.6%) were only osteoporotic, 19/141 (13.5%) were only sarcopenic and 20/141 (14.2%) osteosarcopenic (i.e. both sarcopenia and osteoporosis). The prevalence of osteoporosis was higher in sarcopenic than in non-sarcopenic individuals (51.3% vs. 21.6%, *p* < 0.001). Sarcopenic, osteoporotic and osteosarcopenic subjects had a lower BMI, MNA-SF, handgrip and gait speed (*p* < 0.05) than the reference group (those neither osteoporotic nor sarcopenic, *n* = 80). The Barthel index was lower for sarcopenic and osteosarcopenic (*p* < 0.05) but not for osteoporotic (*p* = 0.07) subjects. The BMI and MNA-SF were lower in osteosarcopenia compared to sarcopenia or osteoporosis alone (*p* < 0.05) while there were no differences in functional criteria.

**Conclusion:**

Osteoporosis and sarcopenia are linked to nutritional deficits and reduced function in geriatric inpatients. Co-occurrence (osteosarcopenia) is common and associated with a higher degree of malnutrition than osteoporosis or sarcopenia alone.

## Zusammenfassung

**Hintergrund:**

Sarkopenie und Osteoporose haben gemeinsame Pathomechanismen und wirken synergistisch bezüglich negativer Gesundheitsoutcomes.

**Ziel der Arbeit:**

Evaluation, in welchem Ausmaß Sarkopenie (nach Definition der European Working Group on Sarcopenia in Older People) und Osteoporose (nach Definition der Weltgesundheitsorganisation) in einem Kollektiv geriatrischer Krankenhauspatienten gleichzeitig vorkommen und mit welchen nutritiven und funktionellen Charakteristika sie verknüpft sind.

**Material und Methoden:**

Wir führten eine Querschnittsanalyse an den Teilnehmern der Sarcopenia-in-Geriatric-Elderly(SAGE)-Studie durch. Gemessen wurden die Knochendichte und Extremitätenmuskelmasse mittels Dualröntgenabsorptiometrie, Gehgeschwindigkeit, Handkraft, Barthel-Index, Body-Mass-Index (BMI) und Mini Nutritional Assessment Short Form (MNA-SF).

**Ergebnisse:**

Von 141 auswertbaren Teilnehmern (84 Frauen, 57 Männer, Durchschnittsalter 80,6± 5,5 Jahre) waren 22 (15,6 %) nur osteoporotisch, 19 (13,5 %) nur sarkopen und 20 (14,2 %) osteosarkopen (sowohl osteoporotisch als auch sarkopen). Die Osteoporoseprävalenz war höher für sarkopene als nichtsarkopene Individuen (51,3 % vs. 21,6 %, *p* <  0,001). Sarkopene, osteoporotische und osteosarkopene Patienten hatten schlechtere BMI-, MNA-SF-, Handkraft- und Gehgeschwindigkeitswerte (*p* <  0,05) als die Kontrollgruppe (weder osteoporotisch noch sarkopen, *n* =  80). Der Barthel-Index war erniedrigt bei den sarkopenen und osteosarkopenen (*p* < 0,05), aber nicht bei den osteoporotischen Teilnehmern (*p* < 0,07). Osteosarkopene Patienten wiesen gegenüber bloß sarkopenen oder osteoporotischen Patienten einen niedrigeren BMI- und MNA-SF-Wert auf (*p* < 0,05), ohne dass sich eine Differenz in funktionellen Kriterien nachweisen ließ.

**Diskussion:**

Osteoporose und Sarkopenie sind bei geriatrischen Krankenhauspatienten mit nutritiven und funktionellen Defiziten und einer reduzierten Funktionsfähigkeit verknüpft. Die Überlappung der beiden Krankheitsentitäten ist häufig. Osteosarkopene Patienten haben einen besonders schlechten Ernährungsstatus.

## Introduction

Sarcopenia and osteoporosis are highly prevalent in old people and contribute to a variety of negative health outcomes [1]. As reflected by the term osteosarcopenia there is an increasing awareness of the interrelationship between muscle and bone disease [1] regarding genetic regulation [2], the endocrine framework [3] and close mechanical interaction [4]. Another common feature of both conditions is structural degradation due to lipotoxicity [5].

Sarcopenia-associated risk of falling and increased bone vulnerability have a synergistic impact on fracture incidence [6]. Despite being named the hazardous duet [7] both conditions are treatable, with a substantial overlap in the options available (exercise, protein supplementation, vitamin D) [8]. While there are some prevalence data from community dwelling elders, in particular fallers [9] and patients with a history of hip fractures [10], data from geriatric hospitals about osteosarcopenic patients are widely lacking.

In order to determine the frequency of co-occurrence of sarcopenia and osteoporosis and the functional and nutritional characteristics accompanying these conditions in geriatric inpatients the dataset of the SAGE study [11] was examined.

## Methods

### Study population

The SAGE study is a cross-sectional study concerned with issues of muscle mass measurement in geriatric inpatients. Preliminary results and study design have been published elsewhere [11]. Briefly the study recruited 148 geriatric inpatients (99 female and 59 male) at the department of Geriatric Medicine, Paracelsus Medical University Salzburg. Inclusion criteria were admittance to a geriatric ward in the study period, ability to walk a few meters and to lie still for 5 min. The lower age limit was 70 years. Exclusion criteria were critical or terminal illness, advanced dementia or delirium, indwelling electrical devices such as pacemakers (bioimpedance analysis being part of the study protocol) and complete or partial amputation of one or more limbs. All participants gave written informed consent. The study was approved by the local ethics committee of the state of Salzburg. For the ancillary osteosarcopenia investigation all SAGE participants with a sufficient dataset were included to enable the diagnosis of sarcopenia and osteoporosis.

### Parameters

#### Baseline characteristics.

In addition to age and gender, the number of comorbidities (out of the nine following: cardiovascular disease, chronic heart failure, cerebrovascular disease, obstructive pulmonary disease, diabetes, renal failure, hypertension, cancer and dementia) were looked into. Besides the number of medications, the life setting (community dwelling vs. institutionalized) and the use of a walking device were assessed. Furthermore, the number of patients in which initial hospital admission was due to fractures was retrieved. Information was obtained from the clinical records.

#### Functional and nutritional parameters.

Gait speed and hand grip strength were measured in all individuals, partly for the diagnosis of sarcopenia but also for comparison between study subgroups. Gait speed was measured over a distance of 5 m. Hand grip strength was measured with a JAMAR^R^ hydraulic hand dynamometer (Ametek, Chicago, IL, USA). A total of six measurements were performed alternating left and right side and the maximum value was selected. The Barthel index at admission and BMI were obtained from the clinical records. The MNA-SF (and if pathologic full MNA) was assessed by a dietician.

#### Diagnosis of sarcopenia.

Diagnosis was based on the EWGSOP criteria, comprising gait speed, handgrip strength and appendicular muscle mass derived from dual energy X‑ray absorptiometry (DXA) [12]. A gait speed of ≤0.8 m/s was considered pathologic. Low hand grip strength was defined as <30 kg for men and <20 kg for women. The Hologic Discovery A (S/N 85001 Hologic Inc. Marlborough, MA, USA) was used for all DXA scans. The scan measurements and analyses were conducted following standard procedures. Participants were measured wearing only gowns to eliminate possible artifacts due to clothing and fasteners. Whole body scans were manually analyzed for the manufacturer-defined regions of interest (ROI) following the standard analysis protocol in the Hologic User Manual. Customized ROI were also analyzed using the Hologic whole body and subregion analysis modes (software ver. 13.3.01). Appendicular skeletal muscle mass (ASMM) was directly derived from the appendicular soft lean tissue compartment in the DXA studies and denoted ASMM_DXA_. For DXA-derived muscle mass the thresholds communicated by Baumgartner et al. [13] were applied based on an appendicular skeletal muscle mass index (ASMMI): ASMMI_DXA_ < 7.26 kg/m^2^ for men and <5.5 kg/m^2^ for women.

#### Diagnosis of osteoporosis.

Bone mineral density (BMD) measurements were performed at three sites in the same session and with same DXA device: lumbar spine (L2–L4), total hip and femoral neck. A T-score of ≤−2.5 standard deviation (SD) at any location was considered diagnostic of osteoporosis [14].

#### Diagnosis of osteosarcopenia.

Osteosarcopenia was diagnosed when both sarcopenia and osteoporosis were present.

#### Data analysis.

The basic pattern of data analysis was to divide the study group into 4 subgroups: 1. sarcopenia without osteoporosis, 2. osteoporosis without sarcopenia, 3. osteosarcopenia (both conditions present) and 4. absence of both conditions (the latter defined as the reference group). For some of the analyses all sarcopenic (with or without osteoporosis) and all osteoporotic (with or without sarcopenia) patients were pooled. Statistical analysis was performed by SPSS^R^ statistics 24. Descriptive values are presented as mean ± standard deviation (±SD). Fisher’s exact test was used for the analysis of qualitative data. Significance of quantitative differences between subgroups was determined by unpaired t‑test. To check for the confounding effect of age and gender multiple regression analysis was used, when means between subgroups were compared. For contingency tables the Mantel-Haenszel method of weighted odds ratios was applied. A *p*-value <0.05 was considered significant.

## Results

Of the 148 geriatric inpatients participating in SAGE, 141 had a dataset allowing the diagnosis of sarcopenia and osteoporosis. Thus 84 women and 57 men (mean age 80.7 ± 5.3 years vs. 80.4 ± 5.8; *p* = 0.73 years) could be included in this investigation. Basic characteristics of the different subgroups of the study population are shown in Table 1. Out of 141 patients 26 (18.4%) had been admitted to hospital with recent fractures.

**Table 1:** Characteristics of the different subgroups of the study population

	OP only (*n* = 22)	SP only (*n* = 19)	OSP (*n* = 20)	OP total (*n* = 42)	SP total (*n* = 39)	RG (*n* = 80)	Total (*n* = 141)
Age (years)	82.5 ± 4.5	81.1 ± 6.3	82.0 ± 6.3	82.3 ± 5.5	81.6 ± 6.3	79.6 ± 3.2	80.6 ± 5.5
Gender (female/male)	20/2	4/15	14/6	34/8	18/21	46/34	84/57
Comorbidities^a^ (m ± SD)	2.5 ± 1.4	3.2 ± 1.4	2.4 ± 1.6	2.5 ± 1.5	2.8 ± 1.6	2.7 ± 1.5	2.7 ± 1.5
Medications (m ± SD)	8.2 ± 3.3	8.2 ± 3.1	7.8 ± 3.9	8.0 ± 3.6	8.0 ± 3.6	8.6 ± 3.2	8.4 ± 3.3
Malnutrition^b^	12	12	17	29	29	37	78
ADL dependency^c^	8	11	15	23	26	32	66
Community dwelling	21	17	16	37	33	76	130
Use of walking aid	16	16	14	30	30	43	89

*OP only* osteoporosis no sarcopenia, *SP only* sarcopenia no osteoporosis, *OSP* sarcopenia and osteoporosis, *OP total* OP only + OSP, *SP total* SP only + OSP, *RG* reference group (no sarcopenia, no osteoporosis), *m* arithmetic mean, *SD* standard deviation, *MNA* mini nutritional assessment, *ADL* activities of daily living

^a^ Selected comorbidities (see methods)

^b^ MNA <17

^c^ Barthel index <70

**Fig. 1:**
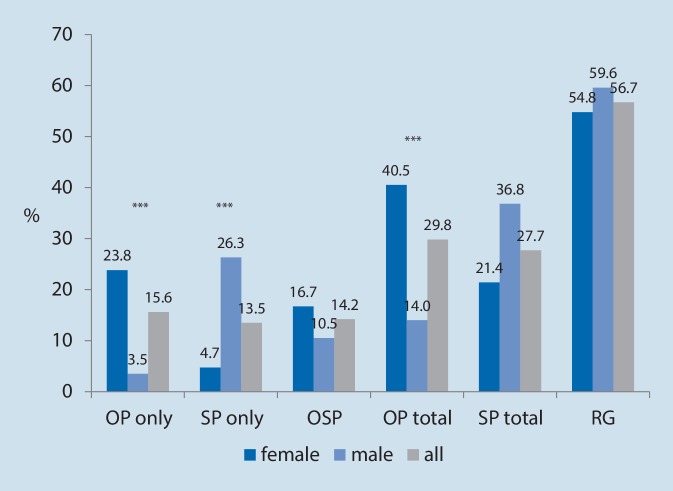
Prevalence of sarcopenia, osteoporosis and osteosarcopenia in the study populations (*OP* osteoporotic; *SP* sarcopenic, *OSP* sarcopenia and osteoporosis, *RG* reference group). Prevalence significantly gender associated (Fisher’s exact test) at *p <0.05, **p <0.01;***p <0.001

Prevalence data are displayed in Fig. 1. Overall prevalences of osteoporosis, sarcopenia and osteosarcopenia were 29.8%, 27.7% and 14.2%, respectively. Osteoporosis was significantly more prevalent among women (40.5% vs. 14.0%, *p* < 0.001), whereas no significant gender difference was observed for sarcopenia and osteosarcopenia. Women had isolated osteoporosis more frequently (*p* < 0.001) while men had isolated sarcopenia more frequently (*p* < 0.001).

**Fig. 2:**
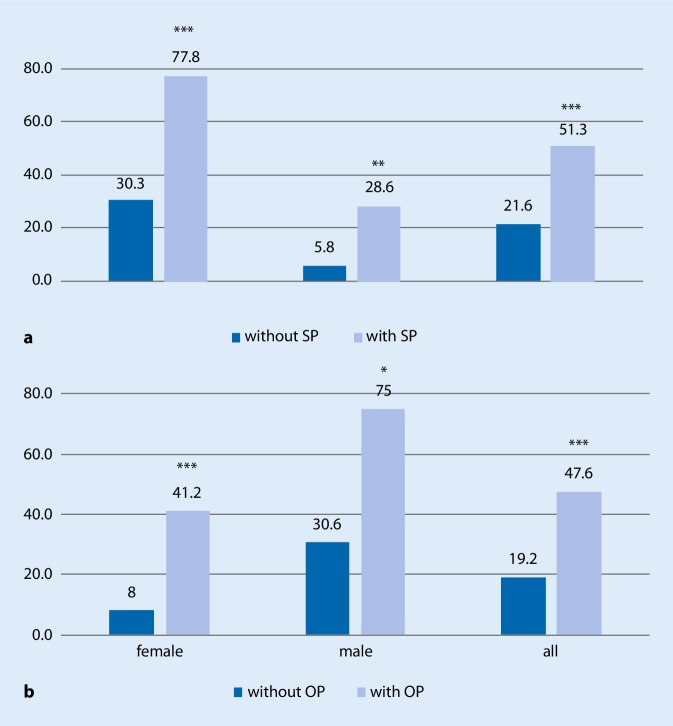
Co-occurrence of sarcopenia and osteoporosis. **a** Prevalence of osteoporosis dependent on sarcopenia status. *SP* sarcopenia. Prevalence significantly different (Fisher’s exact test) at *p <0.05, **p <0.01;***p <0.001 between SP (*lightblue*) and no SP (*dark blue*). **b** Prevalence of sarcopenia depending on osteoporosis status. *OP* osteoporotic. Prevalence significantly different (Fisher’s exact test) at *p <0.05, **p <0.01;***p <0.001 between OP (*light blue*) and no OP (*dark blue*)

There was a highly significant association of sarcopenia and osteoporosis in the whole study group (*p* < 0.001) and in female participants (*p* < 0.001) and a significant association in men (*p* = 0.02) (Fig. 2). The age and gender corrected odds ratio (Mantel-Haenszel test) for the sarcopenic group to be also osteoporotic was 8.71 (confidence interval 2.87–26.42; *p* < 0.001).

The presence of sarcopenia, osteoporosis and osteosarcopenia was associated with nutritional and functional deficits when compared to the reference group (Table 2). All three conditions showed a highly significant association with lower BMI and MNA-SF. In terms of functionality, the Barthel index was lower in the sarcopenic and osteosarcopenic but not in the osteoporotic patients. All three groups had significantly lower gait speed than the reference group. These findings proved statistically robust (*p* < 0.05) when checking for gender and age as possible confounders by multiple regression analysis.

**Table 2:** Functional and nutritional characteristics depending on OS/SP status

	BMI (kg/m^2^)	MNA-SF	HG male (kg)	HG female (kg)	Gait speed (m/s)	Barthel index
RG	28.46 ± 4.15	11.21 ± 2.04	33.65 ± 9.46	21.40 ± 6.57	0.91 ± 0.35	73.56 ± 19.27
OP total	23.68 ± 3.96***	9.69 ± 2.64**	30.17 ± 10.49	16.63 ± 4.42***	0.67 ± 0.24***	66.43 ± 20.33
SP total	23.08 ± 3.52***	9.33 ± 2.44***	28.26 ± 7.99*	16.56 ± 4.13**	0.62 ± 0.20***	61.79 ± 20.58**
OSP	21.46 ± 2.53***	8.50 ± 2.53***	32.25 ± 11.58	16.75 ± 4.43(**)	0.61 ± 0.16***	61.00 ± 15.86**
OP only	25.71 ± 3.95**	10.77 ± 2.25	26.00 ± 6.00	16.53 ± 4.40***	0.72 ± 0.29(*)	71.36 ± 22.57
SP only	24.78 ± 3.61***	10.21 ± 2.02	29.00 ± 5.37**	15.75 ± 2.86(*)	0.62 ± 0.23**	62.63 ± 24.57

Values given as arithmetic mean ± standard deviation

*RG* reference group (no sarcopenia, no osteoporosis), *OP* osteoporotic, *SP* sarcopenic, *OSP* sarcopenia and osteoporosis, *BMI* body mass index, *HG* handgrip, *MNA-SF* mini nutritional assessment short form

Significant at *p <0.05; **p <0.01;***p <0.001 compared to reference group (male and female parts of reference group for handgrip, respectively). Where differences no longer reached statistical significance after correcting for age and gender the asterisks are put into brackets

Pathologically decreased handgrip strength occurred more frequently in all disease subgroups (barely osteoporotic, all osteoporotic, osteosarcopenic, barely sarcopenic and total sarcopenic) than in the reference group (*p* < 0.01). Absolute strength was analyzed separately for female and male participants. Women consistently showed lower grip strength associated with all kinds of muscle and bone disease (*p* < 0.05). This effect persisted after correction for age in all osteoporotic and all sarcopenic women but lost statistical significance for the osteosarcopenic subgroup. No such association was observed in men. Given the small sample size in the different disease subgroups among men, all pathologic conditions were pooled and compared to the male part of the reference group (handgrip 28.05 ± 7.85 kg vs. 33.65 ± 8.08 kg; *p* = 0.017). Pooling of sarcopenic male (sarcopenia only + osteosarcopenia) still showed a significant lower handgrip compared to the male reference group while pooling of osteoporosis (*n* = 2) only and osteosarcopenia (*n* = 6) failed to show a significant difference to the reference group (Table 2). No differences were observed in functional state when the subgroup of osteosarcopenic subjects was compared to sarcopenic and osteoporotic subjects (data not shown); however, BMI and MNA-SF were lower in osteosarcopenic patients when compared to barely sarcopenic and barely osteoporotic respectively (Table 3). This remained true after correction for age and gender.

**Table 3:** Nutritional state and OS/SP status

	BMI [kg/m^2^]	MNA-SF
OSP	21.46 ± 2.53	8.50 ± 2.52
SP only	24.78 ± 3.61**	10.21 ± 2.02*
OP only	25.71 ± 3.95***	10.77 ± 2.25**

Values given as arithmetic mean ± standard deviation

*OSP* sarcopenia and osteoporosis; *OP* all osteoporotic; *SP* all sarcopenic; *BMI* body mass index; *HG* handgrip; *MNA-SF* mini nutritional assessment short form

Significant at *p <0.05; **p <0.01;***p <0.001 compared to OSP group. All differences remained statistically significant after correction for age and gender

## Discussion

In terms of age, comorbidity, polypharmacy and walking limitation (use of walking device) this sample reflected the diversity of hospitalized geriatric patients. The sex ratio (59.6% women) is consistent with the predominance of women on a geriatric ward. The characteristics of this sample match the features of geriatric inpatients as described exemplarily in a cohort study by von Renteln-Kruse and Ebner [16]. The prevalence of osteosarcopenia was 14.2% with a non-significant tendency for higher prevalence (16.7% vs. 10.5%) among women. This is in line with a Chinese study reporting a prevalence of 15.1% (women) and 10.4% (men) in community dwelling elders over age 80 years [15]. Recently Locquet et al. found a prevalence of 9.52% in an exclusively female population (mean age 74.3 years) from Belgium [17]. Some studies [9, 10] reported considerably higher prevalence rates (58% [9], 37.9% [10]) but these referred to preselected populations of female hip fracture patients and fallers, respectively. Osteoporosis was more frequent in sarcopenic (51.3%) than non-sarcopenic (21.6%) subjects. A higher osteoporosis prevalence with respect to sarcopenia status was described in earlier studies (46.1% vs. 22.0% [17], 58.5% vs. 36.4% [9], 57.8% vs. 22.0% [18]) and was seen in both female and male subjects. The nutritional state of all sarcopenic, osteoporotic and osteosarcopenic patients was characterized by lower BMI and MNA-SF compared to the reference group. The BMI and MNA-SF were lowest in osteosarcopenic subjects with a significant difference not only compared to normal but even compared to the barely sarcopenic and barely osteoporotic subjects. A worse nutritional state in osteosarcopenic subjects was also observed by Huo et al. [9] who concluded that thorough nutritional assessment and early supplementation were especially important in this subgroup.

All three pathologic conditions were accompanied by lower gait speed. This was also true for hand grip strength in women and both sexes combined. In men the difference in hand grip yielded statistical significance after pooling the different disease subgroups. A lower Barthel index was associated with sarcopenia and osteosarcopenia but not with osteoporosis. There was no significant difference in functional parameters between the osteosarcopenic and the barely osteoporotic or sarcopenic. Drey et al. [1] reported decreased hand grip strength for the osteosarcopenic subgroup in a population of 68 prefrail individuals but did not find any difference in gait speed; however, this study is not directly comparable due to a different definition of osteosarcopenia which included osteopenic patients with sarcopenia. In a larger (*n* = 680) Australian population of older fallers the osteosarcopenic individuals had decreased hand grip strength as well as gait speed. Previous data pertaining to ADL status in osteosarcopenia could not be found.

There are important limitations to this study. First, due to exclusion of the most frail and demented patients, the population might not entirely reflect a geriatric ward population; however, it is probable that the selection bias is towards underestimation of prevalence rates. Second, the study lacks statistical power especially due to the low number of males with osteoporosis or osteosarcopenia. Larger multicenter studies are needed that allow stratification of the independent contributors to osteosarcopenia. Finally, it is a cross sectional study that does not allow the establishment of chronological or causal relationships in the pathways leading to osteosarcopenia. The strength of the study is to deliver data from a geriatric hospital giving the clinician an idea about the prevalence, the degree of overlap and the associated nutritional and functional deficits of two highly important pathologies of the locomotor and skeletal system.

## Practical conclusion

Osteoporosis and sarcopenia are prevalent conditions on a geriatric ward. Both are related to poor function and malnutrition. Co-occurrence (osteosarcopenia) is frequent and it is associated with a more compromised nutritional state than isolated osteoporosis or sarcopenia. The use of DXA might prove useful in co-diagnosing the two conditions.
